# Association of *IL-10* and *TNF-α* Polymorphisms with Dental Peri-Implant Disease Risk: A Meta-Analysis, Meta-Regression, and Trial Sequential Analysis

**DOI:** 10.3390/ijerph18147697

**Published:** 2021-07-20

**Authors:** Ladan Jamshidy, Santosh Kumar Tadakamadla, Parsia Choubsaz, Masoud Sadeghi, Jyothi Tadakamadla

**Affiliations:** 1 Department of Prosthodontics, School of Dentistry, Kermanshah University of Medical Sciences, Kermanshah 6713954658, Iran; ladanjamshidy@yahoo.com; 2 School of Medicine and Dentistry & Menzies Health Institute Queensland, Griffith University, Brisbane, QLD 4222, Australia; 3 Department of Prosthodontics, School of Dentistry, Shahid Beheshti University of Medical Sciences, Tehran 1983963113, Iran; parsia.choubsaz@sbmu.ac.ir; 4 Medical Biology Research Center, Kermanshah University of Medical Sciences, Kermanshah 6714415185, Iran; sadeghi_mbrc@yahoo.com; 5 School of Medicine and Dentistry, Griffith University, Brisbane, QLD 4222, Australia; j.tadakamadla@griffith.edu.au

**Keywords:** peri-implant disease, implant failure, peri-implantitis, bone loss, cytokine, polymorphism

## Abstract

Genetic susceptibility has been reported to be an important risk factor for peri-implant disease (PID). The aim of this meta-analysis was to assess the association between *TNF-α* and *IL-10* polymorphisms and PID susceptibility. The Web of Science, Cochrane Library, Scopus, and PubMed/Medline databases were searched for studies published until 12 April 2021. RevMan 5.3, CMA 2.0, SPSS 22.0, and trial sequential analysis software were used. Twelve studies were included in our analysis. The pooled ORs for the association of *TNF-α (−308 G > A)*, *IL-10 (−1082 A > G)*, *IL-10 (−819 C > T)*, and *IL-10 (−592 A > C)* polymorphisms were 1.12, 0.93, 1.35, and 0.77 for allelic; 1.42, 0.95, 3.41, and 0.34 for homozygous; 1.19, 1.88, 1.23, and 0.49 for heterozygous, 1.53, 1.12, 1.41, and 0.39 for recessive; and 1.16, 1.87, 2.65, and 0.75 for dominant models, respectively, with all the estimates being insignificant. The results showed an association between *TNF-α (−308 G > A)* polymorphism and the risk of PID in patients of Asian ethnicity (OR = 1.59; *p* = 0.03). The present meta-analysis illustrated that *TNF-α (−308 G > A)*, *IL-10 (−1082 A > G)*, *IL-10 (−819 C > T)*, and *IL-10 (−592 A > C*) polymorphisms were not associated with the risk of PID, whereas *TNF-α (−308 G > A)* polymorphism was associated with an elevated risk of PID in Asian patients.

## 1. Introduction

Despite the high survival rate and success of dental implants, it has long been known that osseointegrated implants may suffer from biological complications, collectively referred to as peri-implant disease (PID) [1]. PIDs are defined as inflammatory lesions of the tissue around the implant and include mucositis around the implant (inflammatory lesion confined to the mucosa around the implant) and peri-implantitis (an inflammatory lesion of the mucosa that affects the supporting bone with bone loss) [2]. A recent meta-analysis included peri-implantitis, implant failure, and marginal bone loss as PIDs [3]. A review study showed peri-implantitis in 28% and ≥56% of cases and in 12% and 43% of implant sites [4]. A systematic review suggested that the prevalence of peri-implantitis was approximately 22% (range: 1–47%) [5]. Another study found the prevalence of dental implant failures to be 11% in males and 9% in females; this prevalence was dependent on implant length, implant diameter, and bone quality [6]. Marginal bone loss (>0.5 mm) at implants was also recognized in 30% of cases and 16% of implant sites [7]. Evidence suggests that those who are aged more than 60 years, smokers, receiving head and neck radiation, postmenopausal, suffering from diabetes, and receiving hormone replacement therapy experienced significantly elevated implant failure in comparison with healthy patients [8].

Genetic susceptibility has been shown to be a significant risk factor for peri-implantitis, and there are numerous studies assessing this in different populations [9,10,11]. Gene polymorphisms refer to changes in DNA sequencing, such as the regulation of inflammatory mediators, primarily the gene promoter region, which can affect gene function and the progression of inflammatory diseases [12,13]. Polymorphisms of cytokines associated with the risk of PID, such as *interleukin (IL)-1A* [14], *IL-1B* [14,15], *IL-6* [16,17], tumor necrosis factor-alpha (*TNF-α*) [17], and *IL-10* [15,18] as an anti-inflammatory cytokine, could inhibit the production of proinflammatory cytokines and the induction of B lymphocyte proliferation as well as prevent the proliferation and activation of natural killer cells [19]. TNF-α is another anti-inflammatory cytokine that plays an important role in inflammatory processes [17]. The role of TNF-α in the destruction of bone around the implant has been suggested by researchers [20]. A meta-analysis [21] assessed the association of *TNF-α (−308 G > A)* and *IL-10 (−1082 A > G)* polymorphisms with the risk of implant failure by including two and three studies, respectively. Another meta-analysis [3] investigated the role of *TNF-α (−308 G > A)* polymorphism in PID using the data from six studies. Their results did not show any association between these polymorphisms and the risk of dental implant failure [21] and PID [3].

The aim of this study was to evaluate the association between *TNF-α (−308 G > A)*, *IL-10 (−1082 A > G)*, *IL-10 (−819 C > T)*, and *IL-10 (−592 A > C)* polymorphisms and PID susceptibility with more studies and the addition of two new polymorphisms (*IL-10 (−819 C > T)* and *IL-10 (−592 A > C)*), meta-regression, and trial sequential analysis (TSA) compared to two previous meta-analyses.

## 2. Materials and Methods

### 2.1. Design

The preferred reporting items for systematic review and meta-analysis (PRISMA) guidelines were used to report this study [22]. The PICO (patient/population, intervention, comparison, and outcomes) question was as follows: Is there an association between *IL-10* and *TNF-α* polymorphisms and the risk of PID in patients with dental implants?

### 2.2. Literature Search Strategy

The Web of Science, Cochrane Library, Scopus, and PubMed/Medline databases were searched for studies published until 12 April 2021 without any restrictions. The searched terms were (“dental implant*” or “oral implant*” or “peri-implant disease*” or “implant loss” or “implant failure” or “peri-implantitis” or “periimplant” or “implant bone loss” or “failing implant”) and (“interleukin-10” or “IL-10” or “IL10” or “TNFA” or “TNF-α” or “TNF-alpha” or “TNFalpha” or “tumor necrosis factor-alpha” or “tumor necrosis factor alpha” or “TNFα” or “tumor necrosis factor-α”) and (“polymorphism*” or “allele” or “genotype*” or “variant*” or “SNP”). In addition, we manually checked the references of seminal articles related to the subject area to ensure that no potential articles were missed.

### 2.3. Eligibility Criteria

The studies were retrieved from the databases by one author (M.S.), and the duplicates and irrelevant studies were then excluded. The studies were considered relevant if they met the following eligibility criteria: (I) case–control design; (II) PID as the outcome of interest; (III) reporting *TNF-α (−308 G > A)*, *IL-10 (−1082 A > G)*, *IL-10 (−819 C > T)*, or *IL-10 (−592 A > C)* polymorphisms with any genetic models; and (IV) having the required data to calculate the odds ratios (ORs) with 95% confidence intervals (CIs) for the genetic models. The studies were removed if they did not have the required data regarding genotype distributions or were animal studies, meta-analyses, review articles, letters to the editor, reported secondary data, and reported genotype distributions after treatment. The second author (L.J.) rechecked the relevant articles based on the eligibility criteria. Any disagreement between the two authors was resolved by discussion.

### 2.4. Data Extraction

One author (M.S.) independently extracted the data from each study and another author (J.T) rechecked them. The information retrieved from the studies included the first author’s name, publication year, ethnic group, control source, mean/median age and male/female ratio in the two groups (patients and controls), genotyping method, form of disease, number of patients or controls, the *p*-value of Hardy–Weinberg equilibrium (HWE) in controls, the quality score, and the distribution of genotypes in the two groups. If there was a disagreement between the authors, the problem was resolved by a short discussion.

### 2.5. Quality of Assessment

One author (L.J) distinguished the quality of each included article using the modified Newcastle–Ottawa Quality Assessment Scale questionnaire, which was used in a similar meta-analysis involving gene polymorphisms. It involves assigning scores ranging from 0–2 and 0–1 on five (representativeness of cases, ascertainment of case outcomes, ascertainment of controls, H–W equilibrium in controls, and association assessment) and two (description of follow-up and genotyping examination) criteria, respectively. A maximum total score of 12 was possible for each study [3].

### 2.6. Statistical Analyses

The Review Manager 5.3 (RevMan 5.3; the Cochrane Collaboration, the Nordic Cochrane Centre, Copenhagen, Denmark) was used to calculate crude odds ratio (OR) and 95% confidence interval (CI) showing the association between *IL-10* and *TNF-α* polymorphisms and dental PID risk in the five genetic models. To evaluate the pooled OR significance, the Z test was applied with a *p* < 0.05. The Cochrane Q test and I^2^ statistic showed the heterogeneity (inconsistency in the polymorphism effect across primary studies). If there was a statistically significant heterogeneity (*p* < 0.1 or I^2^ > 50%), we used a random-effect model (DerSimonian and Laird method) [23], and if there was no significant heterogeneity, a fixed-effect model (Mantel–Haenszel method) [24] was used.

The chi-square test was used to calculate the *p*-value of HWE in the control group of each study, with *p* < 0.05 indicating a deviation from the HWE.

Subgroup, sensitivity, and meta-regression analyses were performed where possible depending on the number of studies available. The subgroup analysis for explanation of heterogeneity based on a priori hypothesis was done for *TNF-α (−308 G > A)* polymorphism according to the ethnicity, control source, disease form, and number of individuals.

The funnel plots were analyzed by the Egger’s and Begg’s tests (with *p*-values < 0.05 indicating statistically significant existence of the publication bias). To evaluate the stability of the pooled results, we used sensitivity analyses (“one study removed” and “cumulative analysis”) for *TNF-α (−308 G > A)* and *IL-10 (−819 C > T)* polymorphisms. The Comprehensive Meta-Analysis version 2.0 (CMA 2.0; Biostat Inc., Englewood, NJ, USA) was used for sensitivity analyses and assessing publication bias. A meta-regression was performed to check the effect of publication year and number of individuals on the pooled results of *TNF-α (−308 G > A*) polymorphism. SPSS version 22.0 software (IBM Corp. Release 2013. IBM SPSS Statistics for Windows, Version 22.0. Armonk, NY: IBM Corp) was used to calculate the results of meta-regression.

Each meta-analysis may create a false-positive or -negative conclusion [25]. Hence, TSA was conducted using TSA software (version 0.9.5.10 beta) (Copenhagen Trial Unit, Centre for Clinical Intervention Research, Rigshospitalet, Copenhagen, Denmark) to reduce these statistical errors [26]. Additionally, a threshold of futility was tested by TSA to earn a conclusion of no effect before reaching the information size. The required information size (RIS) based on an alpha risk of 5%, a beta risk of 20%, and a two-sided boundary type was computed. For those analyses where the Z-curve reached the RIS line or monitored the boundary line or futility area, it was considered that the studies had adequate sample size and their results were valid. Otherwise, it was assumed that the available information was inadequate and more evidence was needed.

## 3. Results

### 3.1. Study Selection

**Figure 1:**
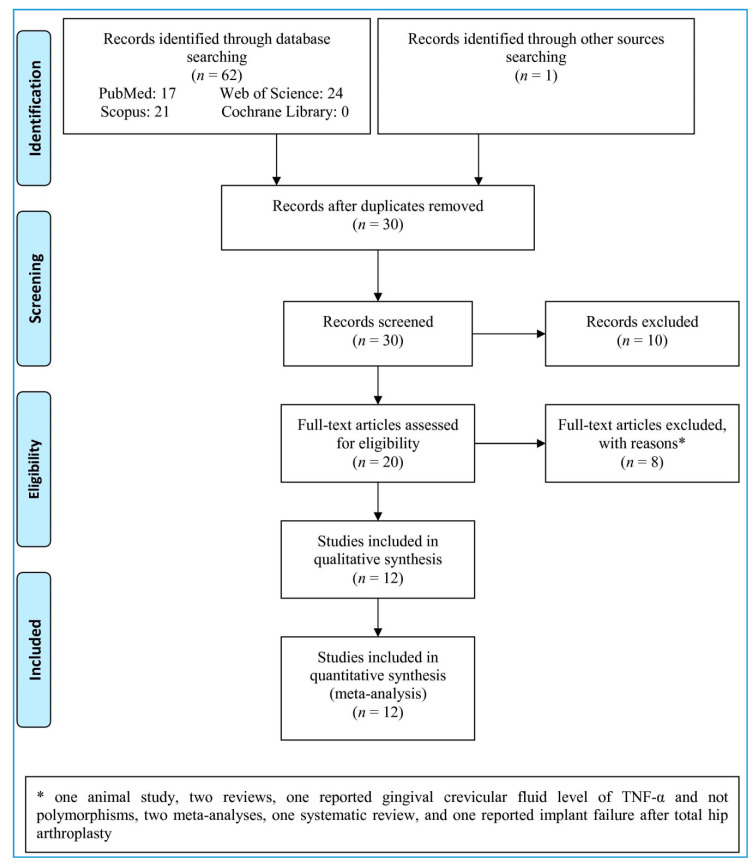
Flow chart of the study selection.

Through the electronic and manual search, 63 records were identified (Figure 1). After removing the duplicates, 30 records were screened, while 10 irrelevant records were removed. A total of 20 full-text articles were evaluated for possible inclusion, and 8 of them were deemed irrelevant and excluded with reasons (one animal study, two reviews, one reported gingival crevicular fluid level of TNF-α and not polymorphisms, two meta-analyses, one systematic review, and one reported implant failure after total hip arthroplasty). Finally, 12 studies were included in our analysis.

### 3.2. Quality Assessment

The quality score for the studies based on modified the Newcastle-Ottawa Scale (NOS) is shown in Table 1. The scores ranged from 8 to 10.

### 3.3. Study Characteristics

Out of the 12 studies [14,15,17,18,27,28,29,30,31,32,33,34], five [15,17,30,31,32] were reported in Caucasian, five [18,27,28,33,34] in mixed race, and two [14,29] in Asian ethnicities (Table 1). The control source was hospital-based in nine studies [17,18,27,28,29,31,32,33,34] and population-based in three studies [14,15,30]. The genotyping method in all studies was based on polymerase chain reaction (PCR). The form of PID in six [18,27,30,31,33,34], five [14,15,17,28,32], and one [29] studies were implant failure, peri-implantitis, and marginal bone loss, respectively.

Table 2 demonstrates the distribution of study population in the included studies based on the genotypes of *TNF-α (−308 G > A)*, *IL-10 (−1082 A > G)*, *IL-10 (−819 C > T)*, and *IL-10 (−592 A > C)* polymorphisms. Ten [14,15,17,27,28,29,30,31,32,34] studies reported genotype prevalence of *TNF-α (−308 G > A)*, four [17,18,30,33] reported *IL-10 (−1082 A > G)*, three [15,18,30] reported *IL-10 (−819 C > T)*, and two [15,18] reported *IL-10 (−592 A > C)* polymorphisms. Among the studies reporting *TNF-α (−308 G > A)* polymorphism, the control group in three studies [14,30,32] had a deviation from HWE. Among the studies reporting *IL-10 (−819 C > T)* polymorphism, one study [30] showed a deviation from HWE for the control group.

### 3.4. Pooled Analyses

The results of meta-analyses based on five genetic models for *TNF-α (−308 G > A)* polymorphism are shown in Table 3. The pooled ORs were 1.12 (95%CI: 0.90–1.39; *p* = 0.32; I^2^ = 43%), 1.42 (95%CI: 0.85–2.37; *p* = 0.18; I^2^ = 0%), 1.19 (95%CI: 0.87–1.63; *p* = 0.28; I^2^ = 0%), 1.53 (95%CI: 0.95–2.45; *p* = 0.08; I^2^ = 59%), and 1.16 (95%CI: 0.74–1.81; *p* = 0.52; I^2^ = 0%) for allelic, homozygous, heterozygous, recessive, and dominant models, respectively. The results showed that *TNF-α (−308 G > A)* polymorphism was not associated with PID risk.

The pooled ORs for allelic, homozygous, heterozygous, recessive, and dominant models of *IL-10 (−1082 A > G)* polymorphism were 0.93 (95%CI: 0.69–1.25; *p* = 0.61; I^2^ = 0%), 0.95 (95%CI: 0.51–1.79; *p* = 0.88; I^2^ = 0%), 1.88 (95%CI: 0.55–1.43; *p* = 0.62; I^2^ = 0%), 1.12 (95%CI: 0.74–1.68; *p* = 0.60; I^2^ = 35%), and 1.87 (95%CI: 0.36–2.11; *p* = 0.76; I^2^ = 56%), respectively (Table 4). The results showed that *IL-10 (−1082 A > G)* polymorphism was not associated with susceptibility to PID.

For allelic, homozygous, heterozygous, recessive, and dominant models of *IL-10 (−819 C > T)* polymorphism, the pooled ORs were 1.35 (95%CI: 1.00–1.82; *p* = 0.05; I^2^ = 37%), 3.41 (95%CI: 0.52–22.17; *p* = 0.20; I^2^ = 60%), 1.23 (95%CI: 0.80–1.90; *p* = 0.35; I^2^ = 0%), 1.41 (95%CI: 0.93–2.13; *p* = 0.10; I^2^ = 0%), and 2.65 (95%CI: 0.53–13.34; *p* = 0.24; I^2^ = 57%), respectively (Table 5). The results reported that there was no association between *IL-10 (−819 C > T)* polymorphism and susceptibility to PID.

Table 6 demonstrates the results for *IL-10 (−592 A > C)* polymorphism with data from two studies for C vs. A, CC vs. AA, AC vs. AA, CC + AC vs. AA, and CC vs. AA + AC genetic models. High heterogeneity was observed in all the models, the pooled ORs were 0.77 (95%CI: 0.18–3.31; *p* = 0.73), 0.34 (95%CI: 0.00–23.53; *p* = 0.62), 0.49 (95%CI: 0.03–9.22; *p* = 0.63), 0.39 (95%CI: 0.01–12.59; *p* = 0.60), and 0.75 (95%CI: 0.14–3.98; *p* = 0.73) for C vs. A, CC vs. AA, AC vs. AA, CC + AC vs. AA, and CC vs. AA + AC, respectively. The results showed that there was no association between *IL-10 (−592 A > C)* polymorphism and susceptibility to PID.

### 3.5. Subgroup Analysis

Subgroup analyses based on ethnicity, control source, disease form, and number of individuals were performed on the association between *TNF-α (−308 G > A)* polymorphism and PID risk (Table 7). The results showed that ethnicity was the only significant factor. Asian patients with *TNF-α (−308 G > A)* polymorphism had a significant elevated risk of PID than the controls (OR = 1.59; *p* = 0.03), whereas there was no significant association between the polymorphism and PID risk for Caucasian and mixed ethnicities.

### 3.6. Sensitivity Analysis

Sensitivity analyses were performed by removing studies with a deviation of HWE in their controls for both *TNF-α (−308 G > A)* and *IL-10 (−819 C > T)* polymorphisms (Table 8). In addition, “one study removed” and “cumulative analyses” were performed, and the results did not change for both the polymorphisms.

### 3.7. Meta-Regression

To check the effect of publication year and sample size on the pooled results of *TNF-α (−308 G > A)* polymorphism, meta-regression was conducted. The findings demonstrated that the publication year and sample size were not confounding factors on the association between *TNF-α (−308 G > A)* polymorphism and susceptibility to PID (Table 9).

### 3.8. Trial Sequential Analysis

**Figure 2:**
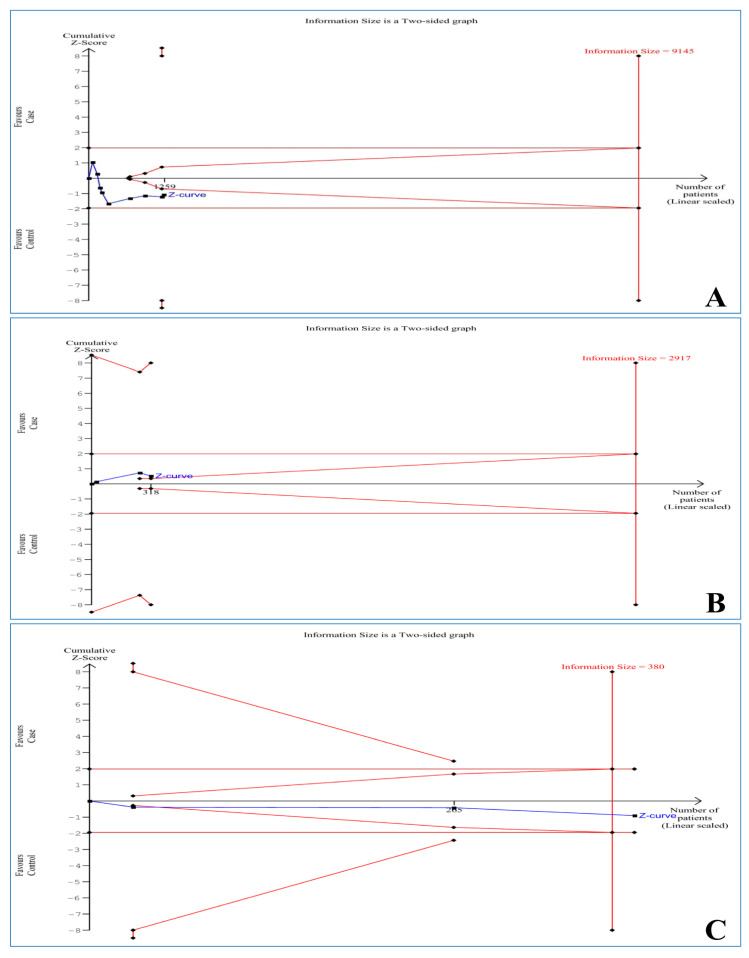
Trial sequential analysis for the association between polymorphisms and dental peri-implant disease risk based on heterozygous model: (**A**) *TNF-α (−308 G > A)*, (**B**) *IL-10 (−1082 A > G)*, and (**C**) *IL-10 (−819 C > T)*.

For *TNF-α (−308 G > A)* and *IL-10 (−1082 A > G)* polymorphisms, the Z-curve did not reach the RIS line or cross the boundary line or enter futility area, establishing that the evidence was not enough for significant results and more information was needed. With regard to *IL-10 (−819 C > T)* polymorphism, the Z-curve exceeded the RIS line, confirming that there was enough evidence to conclude that that the *IL-10 (−819 C > T)* polymorphism was not associated with the PID risk (Figure 2).

### 3.9. Publication Bias

**Figure 3:**
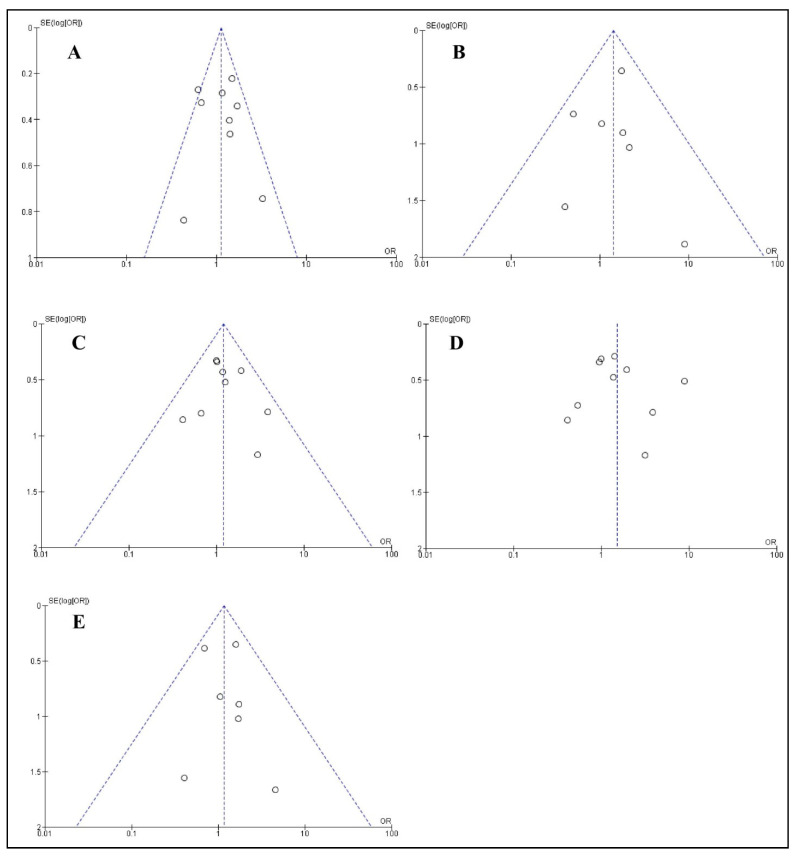
Funnel plot analyses of five genetic models for the association between *TNF-α (−308 G > A)* polymorphism and peri-implant disease risk: (**A**) allelic, (**B**) homozygous, (**C**) heterozygous, (**D**) recessive, and (**E**) dominant.

Funnel plots (Figure 3) along with Egger’s and Begg’s tests demonstrated that there was no publication bias for allelic (Egger’s *p* = 0.859 and Begg’s *p* = 0.834), homozygous (Egger’s *p* = 0.785 and Begg’s *p* = 0.452), heterozygous (Egger’s *p* = 0.667 and Begg’s *p* = 0.835), recessive (Egger’s *p* = 0.633 and Begg’s *p* = 0.929), and dominant (Egger’s *p* = 0.710 and Begg’s *p* = 0.881) models of *TNF-α (−308 G > A)* polymorphism.

## 4. Discussion

Dental implants provide a great treatment option for patients with missing teeth by replacing the root of the tooth with fixed permanent artificial tooth roots that are implanted into the jawbone matching the natural ones and supporting the prosthetic crowns [21].

The main results of the present meta-analysis showed that *TNF-α (−308 G > A)*, *IL-10 (−1082 A > G)*, *IL-10 (−819 C > T)*, and *IL-10 (−592 A > C)* polymorphisms were not associated with PID risk. Out of *TNF-α (−308 G > A)*, *IL-10 (−1082 A > G)*, and *IL-10 (−819 C > T)* polymorphisms, the TSA confirmed the result of only *IL-10 (−819 C > T)* polymorphism, indicating the need for more evidence on *TNF-α (−308 G > A)* and *IL-10 (−1082 A > G)* polymorphisms. The *TNF-α (−308 G > A)* polymorphism had a significant elevated risk in Asian PID patients compared to controls. Moreover, the meta-regression confirmed that publication year and number of individuals were not confounding factors on the association between *TNF-α (−308 G > A)* polymorphism and PID susceptibility.

One research showed increased salivary TNF-α level in cases with peri-implant clinical condition, especially in patients with peri-implantitis [35]. Another research confirmed significantly higher serum level of TNF-α in peri-implantitis patients compared to controls, indicating the pivotal role of these cytokines in peri-implantitis [36]. Farhad et al. [37] concluded that IL-10 level increased in patients with PID compared to individuals with healthy peri-implant tissues, which was also confirmed by many other studies [38,39,40]. Differences in the level of two cytokines between PID patients and controls and the lack of association between the two polymorphisms and the risk of PID in our meta-analysis may indicate the influence of other genetic as well as environmental factors. Future studies might need to explore the influence of these factors.

A meta-analysis examined the association between smoking, radiotherapy, diabetes, and osteoporosis and the risk of dental implant failure [41]. Smoking [17,41,42,43] and radiotherapy [41] are considered the most significant risk factors for dental implant failure. It would be interesting to explore the role of these risk factors on the relationship between gene polymorphism and PID. However, we could not run a meta-regression analysis to assess the effect of these risk factors on the association between gene polymorphisms and PID risk due to unavailability of such data. Wilson and Nunn evaluated the effect of IL-1 polymorphism (smoking and age on dental implant failures) and found that smoking was the only strong risk factor for implant failure [44]. Feloutzis et al. observed similar findings suggesting that IL-1 genotype could further precipitate the detrimental effect of smoking on peri-implant bone loss [45]. Pathogenic bacteria, lack of oral hygiene, and alcohol consumption have also been reported as factors associated with peri-implantitis [42,43]. Research has also indicated the possible effect of systemic diseases on peri-implant bone loss, and most studies therefore recruit PID patients without any systemic diseases [46,47,48,49]. Most studies in our meta-analysis selected individuals who did not smoke or the smoking status was matched between two groups (patients and controls) [14,18,27,28,30,32,33] and without any systemic disease in both cases and controls [18,27,30,33].

Although research exploring the effect of several systemic, habitual, and clinical risk factors on the risk of PID is vast, the effect of genetic risk factors has not been well studied [50,51]. This meta-analyses evaluated *TNF-α (−308 G > A)* and *IL-10 (−1082 A > G)* polymorphisms [21] or *TNF-α (−308 G > A)* polymorphism [3] alone, and no association was observed between any of these polymorphisms and the risk of PID disease. In our meta-analysis, there was an association between *TNF-α (−308 G > A)* polymorphism and PID in Asian patients. We need to further explore the role of ethnicity on the association of the mentioned polymorphisms and PID risk, especially *TNF-α (−308 G > A)* polymorphism.

This meta-analysis had several limitations, namely (1) few studies and lack of subgroup analysis for *IL-10* polymorphisms, (2) smaller sample sizes in some of the included studies, (3) inclusion of smokers as cases and controls in some studies, and (4) the studies that included populations from Asian ethnicity were both from China, meaning the results might not be representative of all Asian population. Lack of publication bias, stability of the pooled data, and the confirmation of the pooled results by TSA would be the important strengths of this meta-analysis.

## 5. Conclusions

The pooled analysis of the present meta-analysis showed that *TNF-α (−308 G > A)*, *IL-10 (−1082 A > G)*, *IL-10 (−819 C > T)*, and *IL-10 (−592 A > C*) polymorphisms were not associated with PID risk, whereas *TNF-α (−308 G > A)* polymorphism was associated with a significant elevated risk of PID in patients of Asian ethnicity.

## Figures and Tables

**Table 1:** Characteristics of studies included in the analysis.

First Author, Publication Year	Country	Ethnicity	Control Source	Case			Control			Genotyping Method	Form of Disease	Quality Score
				Number	Mean/Median Age, Year	Sex (M/F)	Number	Mean/Median Age, Year	Sex (M/F)			
Campos, 2004 [27]	Brazil	Mixed	HB	28	52.7	13/15	38	43.2	18/20	PCR	Implant failure	10
Cury, 2009 [28]	Brazil	Mixed	HB	49	51.1	15/34	41	45.2	17/24	PCR	Peri-implantitis	8
Lu, 2009 [29]	China	Asian	HB	18	47	14/4	26	48	15/11	PCR	Marginal bone loss	8
Gurol, 2011 [30]	Turkey	Caucasian	PB	16	Range: 15–38	-	23	Range: 15–38	-	ARMS-PCR	Implant failure	8
Pigossi, 2012 [18]	Brazil	Mixed	HB	92	55.1	37/55	185	53.1	64/121	RT-PCR	Implant failure	8
Jacobi-Gresser, 2013 [31]	Germany	Caucasian	HB	41	51.1	18/23	68	51.8	16/52	PCR	Implant failure	8
Rakic, 2015 [32]	Serbia	Caucasian	HB	180	53.2	102/78	189	49.4	99/90	PCR-RFLP	Peri-implantitis	10
Petkovic-Curcin, 2017 [17]	Serbia	Caucasian	HB	34	58	26/8	64	58	44/20	PCR-RFLP	Peri-implantitis	8
Ribeiro, 2017 [33]	Brazil	Mixed	HB	29	Range: 21–80	-	61	Range: 21–80	-	ARMS-PCR	Implant failure	8
Broker, 2018 [34]	Brazil	Mixed	HB	81	52.9	30/51	163	51	52/111	RT-PCR	Implant failure	9
He, 2020 [14]	China	Asian	PB	144	45.1	88/56	174	44.3	92/82	PCR	Peri-implantitis	9
Saremi, 2021 [15]	Iran	Caucasian	PB	50	42.2	24/26	89	40.4	43/46	PCR-RFLP	Peri-implantitis	9

HB: hospital-based; PB: population-based; RT-PCR: real-time polymerase chain reaction; ARMS: amplification-refractory mutation system; RFLP: restriction fragment length polymorphism.

**Table 2:** Distribution of the genotypes of four polymorphisms.

First Author, Publication Year	*TNF-α (−308 G > A)*						*p-*Value of HWE in Control
	Case			Control			
	GG	GA	AA	GG	GA	AA	
Campos, 2004 [27]	26	2	0	32	6	0	0.597
Cury, 2009 [28]	34	11	4	31	8	2	0.161
Lu, 2009 [29]	12	6	0	23	3	0	0.746
Gurol, 2011 [30]	1	14	1	4	19	0	< 0.001
Jacobi-Gresser, 2013 [31]	22	17	2	47	19	2	0.962
Rakic, 2015 [32]	157	20	3	165	21	3	0.026
Petkovic-Curcin, 2017 [17]	15	19		56	8		NA
Broker, 2018 [34]	63	16	0	128	32	2	1.000
He, 2020 [14]	113	11	20	146	12	16	< 0.001
Saremi, 2021 [15]	4	12	34	4	18	67	0.074
	*IL-10 (−1082 A > G)*						
	AA	AG	GG	AA	AG	GG	
Gurol, 2011 [30]	2	9	4	3	15	4	0.086
Pigossi, 2012 [18]	36	41	15	65	90	24	0.412
Petkovic-Curcin, 2017 [17]	6	28		25	39		NA
Ribeiro, 2017 [33]	6	16	7	11	24	26	0.204
	*IL-10 (−819 C > T)*						
	CC	CT	TT	CC	CT	TT	
Gurol, 2011 [30]	0	12	1	1	19	1	< 0.001
Pigossi, 2012 [18]	37	38	11	82	76	19	0.824
Saremi, 2021 [15]	22	21	7	53	35	1	0.067
		*IL-10 (−592 A > C)*					
	AA	AC	CC	AA	AC	CC	
Pigossi, 2012 [18]	24	38	12	87	77	18	0.873
Saremi, 2021 [15]	8	26	16	1	35	53	0.067

**Table 3:** The results of meta-analyses based on five genetic models for *TNF-α (−308 G > A)* polymorphism.

Genetic Model	First Author, Publication Year	Case		Control		Weight	Odds Ratio
		Events	Total	Events	Total		M–H, Fixed, 95%CI
A vs. G	Campos, 2004 [27]	2	56	6	76	3.3%	0.43 [0.08, 2.23]
	Cury, 2009 [28]	19	98	12	82	7.0%	1.40 [0.64, 3.09]
	Lu, 2009 [29]	6	36	3	52	1.4%	3.27 [0.76, 14.04]
	Gurol, 2011 [30]	16	32	19	46	5.2%	1.42 [0.57, 3.52]
	Jacobi-Gresser, 2013 [31]	21	82	23	136	8.5%	1.69 [0.87, 3.30]
	Rakic, 2015 [32]	26	324	27	388	15.0%	1.17 [0.67, 2.04]
	Broker, 2018 [34]	26	360	36	324	23.3%	0.62 [0.37, 1.06]
	He, 2020 [14]	51	288	44	348	21.8%	1.49 [0.96, 2.30]
	Saremi, 2021 [15]	80	100	152	178	14.5%	0.68 [0.36, 1.30]
Subtotal (95%CI)			1376		1630	100.0%	1.12 [0.90, 1.39]
Total events		247		322			
Heterogeneity: Chi^2^ = 14.03, df = 8 (*p* = 0.08); I^2^ = 43% Test for overall effect: Z = 1.00 (*p* = 0.32)							
AA vs. GG	Campos, 2004 [27]	0	26	0	32		Not estimable
	Cury, 2009 [28]	0	12	0	23		Not estimable
	Lu, 2009 [29]	4	38	2	33	7.9%	1.82 [0.31, 10.66]
	Gurol, 2011 [30]	1	2	0	4	0.8%	9.00 [0.22, 362.48]
	Jacobi-Gresser, 2013 [31]	2	24	2	49	5.0%	2.14 [0.28, 16.17]
	Rakic, 2015 [32]	3	160	3	168	11.8%	1.05 [0.21, 5.28]
	Broker, 2018 [34]	0	63	2	130	6.7%	0.40 [0.02, 8.56]
	He, 2020 [14]	20	123	16	162	47.6%	1.77 [0.88, 3.58]
	Saremi, 2021 [15]	34	38	67	71	20.2%	0.51 [0.12, 2.15]
Subtotal (95%CI)			486		672	100.0%	1.42 [0.85, 2.37]
Total events		64		92			
Heterogeneity: Chi^2^ = 4.30, df = 6 (*p* = 0.64); I^2^ = 0% Test for overall effect: Z = 1.33 (*p* = 0.18)							
GA vs. GG	Campos, 2004 [27]	2	28	6	38	6.7%	0.41 [0.08, 2.21]
	Cury, 2009 [28]	11	45	8	39	9.2%	1.25 [0.45, 3.52]
	Lu, 2009 [29]	6	18	3	26	2.3%	3.83 [0.81, 18.09]
	Gurol, 2011 [30]	14	15	19	23	1.4%	2.95 [0.30, 29.32]
	Jacobi-Gresser, 2013 [31]	17	39	19	66	11.3%	1.91 [0.84, 4.37]
	Rakic, 2015 [32]	20	177	21	186	25.9%	1.00 [0.52, 1.92]
	Broker, 2018 [34]	16	79	32	160	24.0%	1.02 [0.52, 1.99]
	He, 2020 [14]	11	124	12	158	13.7%	1.18 [0.50, 2.78]
	Saremi, 2021 [15]	12	16	18	22	5.4%	0.67 [0.14, 3.19]
Subtotal (95% CI)			541		718	100.0%	1.19 [0.87, 1.63]
Total events		109		138			
Heterogeneity: Chi^2^ = 6.60, df = 8 (*p* = 0.58); I^2^ = 0% Test for overall effect: Z = 1.09 (*p* = 0.28)							
AA + GA vs. GG	Campos, 2004 [27]	2	28	6	38	5.6%	0.41 [0.08, 2.21]
	Cury, 2009 [28]	15	49	10	41	11.0%	1.37 [0.54, 3.49]
	Lu, 2009 [29]	6	18	3	26	6.3%	3.83 [0.81, 18.09]
	Gurol, 2011 [30]	15	16	19	23	3.5%	3.16 [0.32, 31.29]
	Jacobi-Gresser, 2013 [31]	19	41	21	68	12.5%	1.93 [0.87, 4.31]
	Rakic, 2015 [32]	23	180	24	189	14.6%	1.01 [0.55, 1.86]
	Petkovic-Curcin, 2017 [17]	19	34	8	64	10.4%	8.87 [3.25, 24.19]
	Broker, 2018 [34]	16	79	34	162	14.0%	0.96 [0.49, 1.86]
	He, 2020 [14]	31	144	28	174	15.2%	1.43 [0.81, 2.52]
	Saremi, 2021 [15]	46	50	85	89	7.0%	0.54 [0.13, 2.26]
Subtotal (95%CI)			639		874	100.0%	1.53 [0.95, 2.45]
Total events		192		238			
Heterogeneity: Tau^2^ = 0.30; Chi^2^ = 21.81, df = 9 (*p* = 0.009); I^2^ = 59% Test for overall effect: Z = 1.75 (*p* = 0.08)							
AA vs. GG + GA	Campos, 2004 [27]	0	28	0	38		Not estimable
	Cury, 2009 [28]	0	18	0	26		Not estimable
	Lu, 2009 [29]	4	49	2	41	5.5%	1.73 [0.30, 9.98]
	Gurol, 2011 [30]	1	16	0	23	1.0%	4.55 [0.17, 118.99]
	Jacobi-Gresser, 2013 [31]	2	41	2	68	4.0%	1.69 [0.23, 12.50]
	Rakic, 2015 [32]	3	180	3	189	7.9%	1.05 [0.21, 5.28]
	Broker, 2018 [34]	0	79	2	162	4.5%	0.40 [0.02, 8.51]
	He, 2020 [14]	20	144	16	174	34.4%	1.59 [0.79, 3.20]
	Saremi, 2021 [15]	34	50	67	89	42.6%	0.70 [0.32, 1.50]
Subtotal (95%CI)			605		810	100.0%	1.16 [0.74, 1.81]
Total events		64		92			
Heterogeneity: Chi^2^ = 3.98, df = 6 (*p* = 0.68); I^2^ = 0% Test for overall effect: Z = 0.64 (*p* = 0.52)							

**Table 4:** Results on the association of the five genetic models of *IL-10 (−1082 A > G)* polymorphism with the risk of PID.

Genetic Model	First Author, Publication Year	Case		Control		Weight	Odds Ratio
		Events	Total	Events	Total		M–H, Fixed, 95%CI
G vs. A	Gurol, 2011 [30]	17	30	23	44	9.1%	1.19 [0.47, 3.04]
	Pigossi, 2012 [18]	71	184	138	358	64.5%	1.00 [0.70, 1.44]
	Ribeiro, 2017 [33]	30	58	76	122	26.5%	0.65 [0.34, 1.22]
Subtotal (95%CI)			272		524	100.0%	0.93 [0.69, 1.25]
Total events		118		237			
Heterogeneity: Chi^2^ = 1.68, df = 2 (*p* = 0.43); I^2^ = 0% Test for overall effect: Z = 0.51 (*p* = 0.61)							
GG vs. AA	Gurol, 2011 [30]	4	6	4	7	6.2%	1.50 [0.16, 14.42]
	Pigossi, 2012 [18]	15	51	24	89	62.3%	1.13 [0.53, 2.42]
	Ribeiro, 2017 [33]	7	13	26	37	31.5%	0.49 [0.13, 1.81]
Subtotal (95%CI)			70		133	100.0%	0.95 [0.51, 1.79]
Total events		26		54			
Heterogeneity: Chi^2^ = 1.33, df = 2 (*p* = 0.51); I^2^ = 0% Test for overall effect: Z = 0.15 (*p* = 0.88)							
AG vs. AA	Gurol, 2011 [30]	9	11	15	18	5.9%	0.90 [0.13, 6.46]
	Pigossi, 2012 [18]	41	77	90	155	79.7%	0.82 [0.47, 1.43]
	Ribeiro, 2017 [33]	16	22	24	35	14.4%	1.22 [0.38, 3.97]
Subtotal (95% CI)			110		208	100.0%	0.88 [0.55, 1.43]
Total events		66		129			
Heterogeneity: Chi^2^ = 0.36, df = 2 (*p* = 0.84); I^2^ = 0% Test for overall effect: Z = 0.50 (*p* = 0.62)							
GG + AG vs. AA	Gurol, 2011 [30]	13	15	19	22	4.7%	1.03 [0.15, 7.02]
	Pigossi, 2012 [18]	56	92	114	179	69.2%	0.89 [0.53, 1.49]
	Petkovic-Curcin, 2017 [17]	23	29	50	61	15.2%	0.84 [0.28, 2.56]
	Ribeiro, 2017 [33]	28	34	39	64	10.9%	2.99 [1.08, 8.25]
Subtotal (95%CI)			170		326	100.0%	1.12 [0.74, 1.68]
Total events		120		222			
Heterogeneity: Chi^2^ = 4.64, df = 3 (*p* = 0.20); I^2^ = 35% Test for overall effect: Z = 0.53 (*p* = 0.60)							
GG vs. AA + AG	Gurol, 2011 [30]	4	15	4	22	20.8%	1.64 [0.34, 7.91]
	Pigossi, 2012 [18]	15	92	24	179	44.1%	1.26 [0.62, 2.54]
	Ribeiro, 2017 [33]	7	34	26	64	35.2%	0.38 [0.14, 1.00]
Subtotal (95%CI)			141		265	100.0%	0.87 [0.36, 2.11]
Total events		26		54			
Heterogeneity: Tau^2^ = 0.33; Chi^2^ = 4.50, df = 2 (*p* = 0.11); I^2^ = 56% Test for overall effect: Z = 0.31 (*p* = 0.76)							

**Table 5:** Meta-analyses of studies involving five genetic models of *IL-10 (−819 C > T)* polymorphism and the risk of PID.

Genetic Model	First Author, Publication Year	Case		Control		Weight	Odds Ratio
		Events	Total	Events	Total		M–H, Fixed, 95%CI
T vs. C	Gurol, 2011 [30]	14	26	21	42	10.1%	1.17 [0.44, 3.11]
	Pigossi, 2012 [18]	60	172	114	354	66.3%	1.13 [0.77, 1.66]
	Saremi, 2021 [15]	35	100	37	178	23.6%	2.05 [1.19, 3.55]
Subtotal (95%CI)			298		574	100.0%	1.35 [1.00, 1.82]
Total events		109		172			
Heterogeneity: Chi^2^ = 3.17, df = 2 (*p* = 0.20); I^2^ = 37% Test for overall effect: Z = 1.97 (*p* = 0.05)							
TT vs. CC	Gurol, 2011 [30]	1	1	1	2	16.3%	3.00 [0.06, 151.19]
	Pigossi, 2012 [18]	11	48	19	101	51.2%	1.28 [0.56, 2.97]
	Saremi, 2021 [15]	7	29	1	54	32.5%	16.86 [1.96, 145.27]
Subtotal (95%CI)			78		157	100.0%	3.41 [0.52, 22.17]
Total events		19		21			
Heterogeneity: Tau^2^ = 1.60; Chi^2^ = 4.99, df = 2 (*p* = 0.08); I^2^ = 60% Test for overall effect: Z = 1.28 (*p* = 0.20)							
CT vs. TT	Gurol, 2011 [30]	12	12	19	20	1.6%	1.92 [0.07, 51.03]
	Pigossi, 2012 [18]	38	75	76	158	66.2%	1.11 [0.64, 1.92]
	Saremi, 2021 [15]	21	43	35	88	32.2%	1.45 [0.69, 3.01]
Subtotal (95% CI)			130		266	100.0%	1.23 [0.80, 1.90]
Total events		71		130			
Heterogeneity: Chi^2^ = 0.40, df = 2 (*p* = 0.82); I^2^ = 0% Test for overall effect: Z = 0.93 (*p* = 0.35)							
TT + CT vs. CC	Gurol, 2011 [30]	13	13	20	21	1.5%	1.98 [0.07, 52.16]
	Pigossi, 2012 [18]	49	86	95	177	70.0%	1.14 [0.68, 1.92]
	Saremi, 2021 [15]	29	50	36	89	28.5%	2.03 [1.01, 4.11]
Subtotal (95%CI)			149		287	100.0%	1.41 [0.93, 2.13]
Total events		91		151			
Heterogeneity: Chi^2^ = 1.71, df = 2 (*p* = 0.43); I^2^ = 0% Test for overall effect: Z = 1.63 (*p* = 0.10)							
TT vs. CC + CT	Gurol, 2011 [30]	1	13	1	21	20.5%	1.67 [0.10, 29.18]
	Pigossi, 2012 [18]	11	86	19	177	50.6%	1.22 [0.55, 2.69]
	Saremi, 2021 [15]	7	50	1	89	28.9%	14.33 [1.71, 120.16]
Subtotal (95%CI)			149		287	100.0%	2.65 [0.53, 13.34]
Total events		19		21			
Heterogeneity: Tau^2^ = 1.18; Chi^2^ = 4.69, df = 2 (*p* = 0.10); I^2^ = 57% Test for overall effect: Z = 1.18 (*p* = 0.24)							

**Table 6:** Meta-analyses of association between *IL-10 (−592 A > C)* polymorphism and PID risk based on five genetic models.

Genetic Model	First Author, Publication Year	Case		Control		Weight	Odds Ratio
		Events	Total	Events	Total		M–H, Random, 95%CI
C vs. A	Pigossi, 2012 [18]	62	148	113	364	50.8%	1.60 [1.08, 2.38]
	Saremi, 2021 [15]	58	100	141	178	49.2%	0.36 [0.21, 0.62]
Subtotal (95%CI)			248		542	100.0%	0.77 [0.18, 3.31]
Total events		120		254			
Heterogeneity: Tau^2^ = 1.05; Chi^2^ = 19.07, df = 1 (*p* < 0.0001); I^2^ = 95% Test for overall effect: Z = 0.35 (*p* = 0.73)							
CC vs. AA	Pigossi, 2012 [18]	12	36	18	105	52.7%	2.42 [1.02, 5.70]
	Saremi, 2021 [15]	16	24	53	54	47.3%	0.04 [0.00, 0.32]
Subtotal (95%CI)			60		159	100.0%	0.34 [0.00, 23.53]
Total events		28		71			
Heterogeneity: Tau^2^ = 8.70; Chi^2^ = 13.45, df = 1 (*p* = 0.0002); I^2^ = 93% Test for overall effect: Z = 0.50 (*p* = 0.62)							
AC vs. AA	Pigossi, 2012 [18]	38	62	77	164	56.0%	1.79 [0.99, 3.25]
	Saremi, 2021 [15]	26	34	35	36	44.0%	0.09 [0.01, 0.79]
Subtotal (95% CI)			96		200	100.0%	0.49 [0.03, 9.22]
Total events		64		112			
Heterogeneity: Tau^2^ = 3.93; Chi^2^ = 7.11, df = 1 (*p* = 0.008); I^2^ = 86% Test for overall effect: Z = 0.48 (*p* = 0.63)							
CC + AC vs. AA	Pigossi, 2012 [18]	50	74	95	182	54.3%	1.91 [1.08, 3.36]
	Saremi, 2021 [15]	42	50	88	89	45.7%	0.06 [0.01, 0.49]
Subtotal (95%CI)			124		271	100.0%	0.39 [0.01, 12.59]
Total events		92		183			
Heterogeneity: Tau^2^ = 5.70; Chi^2^ = 10.16, df = 1 (*p* = 0.001); I^2^ = 90% Test for overall effect: Z = 0.53 (*p* = 0.60)							
CC vs. AA + AC	Pigossi, 2012 [18]	12	74	18	182	49.6%	1.76 [0.80, 3.87]
	Saremi, 2021 [15]	16	50	53	89	50.4%	0.32 [0.15, 0.66]
Subtotal (95%CI)			124		271	100.0%	0.75 [0.14, 3.98]
Total events		28		71			
Heterogeneity: Tau^2^ = 1.31; Chi^2^ = 9.75, df = 1 (*p* = 0.002); I^2^ = 90% Test for overall effect: Z = 0.34 (*p* = 0.73)							

**Table 7:** Subgroup analyses based on ethnicity, control source, disease form, and sample size for five genetic models of *TNF-α (−308 G > A)* polymorphism.

Variable (N, N′)	A vs. G	AA vs. GG	GA vs. GG	AA + GA vs. GG	AA vs. GG + GA
	OR (95%CI), *p*, I^2^	OR (95%CI), *p*, I^2^	OR (95%CI), *p*, I^2^	OR (95%CI), *p*, I^2^	OR (95%CI), *p*, I^2^
All (9, 10)	1.12 (0.90, 1.39), 0.32, 43%	1.42 (0.85, 2.37), 0.18, 0%	1.19 (0.87, 1.63), 0.28, 0%	1.53 (0.95, 2.45), 0.08, 59%	1.16 (0.74, 1.81), 0.52, 0%
Ethnicity					
Caucasian (4,5)	1.14 (0.82, 1.59), 0.44, 25%	1.06 (0.43, 2.62), 0.89, 0%	1.26 (0.79, 2.01), 0.34, 0%	1.92 (0.76, 4.89), 0.17, 75%	0.89 (0.47, 1.68), 0.72, 0%
Asian (2, 2)	1.59 (1.05, 2.42), 0.03, 3% *	1.77 (0.88, 3.58), 0.11	1.57 (0.75, 3.27), 0.23, 41%	1.61 (0.95, 2.74), 0.08, 27%	1.59 (0.79, 3.20), 0.19
Mixed (3, 3)	0.77 (0.51, 1.16), 0.21, 40%	1.17 (0.29, 4.81), 0.83, 0%	0.97 (0.57, 1.64), 0.91, 0%	0.97 (0.59, 1.62), 0.92, 0%	1.14 (0.28, 4.63), 0.86, 0%
Control source					
Hospital-based (6, 7)	1.06 (0.79, 1.42), 0.68, 49%	1.28 (0.50, 3.28), 0.61, 0%	1.20 (0.84, 1.71), 0.32, 9%	1.67 (0.89, 3.13), 0.11, 0.69%	1.20 (0.47, 3.07), 0.70, 0%
Population-based (3, 3)	1.20 (0.86, 1.68), 0.29, 50%	1.48 (0.80, 2.74), 0.21, 39%	1.17 (0.58, 2.36), 0.66, 0%	1.33 (0.80, 2.21), 0.28, 6%	1.14 (0.69, 1.90), 0.60, 37%
Disease form					
Peri-implantitis (4, 5)	1.19 (0.90, 1.59), 0.22, 25%	1.39 (0.80, 2.42), 0.25, 0%	1.06 (0.68, 1.65), 0.81, 0%	1.60 (0.77, 3.35), 0.21, 75%	1.13 (0.71, 1.81), 0.60, 0%
Implant failure (4, 4)	0.98 (0.53, 1.83), 0.96, 57%	1.63 (0.41, 6.40), 0.48, 0%	1.22 (0.76, 1.96), 0.41, 17%	1.20 (0.75, 1.91), 0.44, 26%	1.39 (0.36, 5.39), 0.63, 0%
Marginal bone loss (1, 1)	3.27 (0.76, 14.04), 0.11	-	3.83 (0.81, 18.09), 0.09	3.83 (0.81, 18.09), 0.09	-
Number of individuals					
>100 (5, 5)	1.05 (0.71, 1.56), 0.81, 60%	1.32 (0.76, 2.28), 0.32, 0%	1.14 (0.80, 1.63), 0.46, 0%	1.19 (0.87, 1.63), 0.28, 0%	1.09 (0.68, 1.73), 0.73, 0%
≤100 (4, 5)	1.37 (0.82, 2.28), 0.23, 8%	2.46 (0.51, 11.85), 0.26, 0%	1.39 (0.70, 2.77), 0.34, 27%	2.37 (0.80, 7.03), 0.12, 68%	2.18 (0.47, 10.06), 0.32, 0%

* *p* < 0.05.

**Table 8:** Sensitivity analyses removing the studies with a deviation of HWE in their controls for *TNF-α (−308 G > A)* and *IL-10 (−819 C > T)* polymorphisms.

Polymorphism (N, N′)	Allelic	Homozygous	Heterozygous	Recessive	Dominant
	OR (95%CI), *p*, I^2^	OR (95%CI), *p*, I^2^	OR (95%CI), *p*, I^2^	OR (95%CI), *p*, I^2^	OR (95%CI), *p*, I^2^
***TNF-α*** (−308 G > A) (6, 7)	1.02 (0.62, 1.66), 0.95, 54%	0.95 (0.39, 2.35), 0.92, 0%	1.24 (0.82, 1.85), 0.31, 12%	1.61 (0.78, 3.32), 0.20, 70%	0.84 (0.45, 1.60), 0.60, 0%
***IL-10*** (−819 C > T) (2)	1.47 (0.82, 2.64), 0.19, 67%	3.84 (0.30, 48.54), 0.30, 80%	1.22 (0.78, 1.89), 0.38, 0%	1.40 (0.92, 2.12), 0.11, 40%	3.43 (0.30, 38.86), 0.32, 79%

**Table 9:** Meta-regression for *TNF-α (−308 G > A)* polymorphism based on publication year and sample size.

Variable		A vs. G	AA vs. GG	GA vs. GG	AA + GA vs. GG	AA vs. GG + GA
Year of publication	R	0.211	0.522	0.272	0.075	0.585
	Adjusted R^2^	−0.092	0.127	−0.058	−0.119	0.210
	*p*-value	0.586	0.229	0.479	0.837	0.168
Number of individuals	R	0.272	0.558	0.472	0.337	0.566
	Adjusted R^2^	−0.058	0.173	0.112	0.003	0.185
	*p*-value	0.479	0.193	0.200	0.341	0.185

## Data Availability

The data that support the findings of this study are available on request from the corresponding author.
